# Stigma in Male Depression and Suicide: A Canadian Sex Comparison Study

**DOI:** 10.1007/s10597-015-9986-x

**Published:** 2016-01-05

**Authors:** John L. Oliffe, John S. Ogrodniczuk, Susan J. Gordon, Genevieve Creighton, Mary T. Kelly, Nick Black, Corey Mackenzie

**Affiliations:** School of Nursing, University of British Columbia, 109 - 2176 Health Sciences Mall, Vancouver, BC V6T 1Z3 Canada; Department of Psychiatry, University of British Columbia, #420, 5950 University Boulevard, Vancouver, BC V6T 1Z3 Canada; School of Health Sciences, Flinders University, Sturt Road, Bedford Park, Adelaide, SA 5042 Australia; School of Nursing, University of British Columbia, 2176 Health Sciences Mall, Vancouver, BC V6T 1Z3 Canada; Intensions Consulting Inc., Vancouver, BC Canada; Department of Psychology, University of Manitoba, P516 Duff Roblin Bldg, 190 Dysart Road, Winnipeg, MB R3T 2N2 Canada

**Keywords:** Men’s depression, Men’s suicide, Stigma, Self-stigma

## Abstract

Stigma in men’s depression and suicide can restrict help-seeking, reduce treatment compliance and deter individuals from confiding in friends and family. In this article we report sex comparison findings from a national survey of English-speaking adult Canadians about stigmatized beliefs concerning male depression and suicide. Among respondents without direct experience of depression or suicide (n = 541) more than a third endorsed the view that men with depression are unpredictable. Overall, a greater proportion of males endorsed stigmatizing views about male depression compared to female respondents. A greater proportion of female respondents endorsed items indicating that men who suicide are disconnected, lost and lonely. Male and female respondents with direct personal experience of depression or suicide (n = 360) strongly endorsed stigmatizing attitudes toward themselves and a greater proportion of male respondents indicated that they would be embarrassed about seeking help for depression.

## Introduction

Fewer men than women are formally diagnosed with depression, and in Western countries, rates of male depression are half that of females. Experts suggest that the lower reported rates of men’s depression are due, in part, to men’s reluctance to express concerns about their mental health and reticence to seek professional mental health care services (Oliffe and Phillips 2008). Confounding this, male suicide rates are three times higher than that of females (Statistics Canada 2014). Implicated in the discordant relationship between men’s low rates of diagnosed depression and high suicide rates is stigma around mental illness, which can impede men’s help-seeking and/or treatment compliance and limit their self-disclosure about depressive symptoms and/or suicidal thoughts (Livingston and Boyd 2010).

Stigma in mental illness is diverse in terms of how it is defined, operationalized and reported. Personal or internal stigma has been defined as the perception of self as inadequate, due to a mental illness, leading to the loss of self-esteem (Vogel et al. 2006). Public or external stigma refers to negative stereotypes that individuals and communities in a society hold about and/or invoke on persons experiencing mental illness (Corrigan and Watson 2002). In previous work addressing sex differences and mental health, several studies report that males tend to have more negative attitudes toward depression than females (Cook and Wang 2010; Wang et al. 2007). For example, in a survey of 3047 adults, Wang et al. (2007a) found that men (47.2 %) were more likely than women (39.2 %) to attribute “weakness of character” as a probable trigger for depression. Among urban and rural based respondents, rural men had higher stigma toward depression, even more so when they had poor depression literacy (Jones et al. 2011). Men who were unsure about the best available resources for depression or preferred to rely on personal support systems to treat depression were more likely to stigmatize depression (Wang et al. 2007a).

Some research suggests that men with personal experience of depression have higher self-stigma than women (Cook and Wang 2010; Wang et al. 2007b). Fogel and Ford (2005) found no sex differences in stigma toward family members with depression, while other researchers concluded that personally knowing someone with depression was associated with lower stigma scores for women, but not for men (Wang et al. 2007a; Wang and Lai 2008). In a US study of 5,251 adults, 40 % of respondents indicated that they believed people with mental illness were “unpredictable” and 23 % believed that persons with mental illness were “dangerous to others” (Kobau et al. 2010). The male respondents scored slightly higher for stereotypical beliefs about mental illness and more negative attitudes regarding recovery than did female respondents (Kobau et al. 2010). An Australian study investigating public attitudes (n = 6019) toward mental illness reported that stigmatized attitudes were more often attributed to men experiencing mental illness compared to women (Reavley and Jorm 2011). Specifically, men with depression were perceived as “best avoided” by 40 % of respondents, while 50 % of respondents indicated that men with suicidal thoughts were likely to be dangerous (Reavley and Jorm 2011). Stigma and men’s depression work has also highlighted men’s reticence for seeking professional mental health care. In a study of men experiencing depression by Johnson et al. (2012), participants conveyed feeling judged as a major impediment to seeking professional care for depression. Roy et al. (2014) added to this finding, suggesting that men’s help-seeking for professional mental health services was perceived more favorably when an individual believed he had exhausted personal support systems. Media portrayals of male depression that are not representative of the average man can also increase stigma (Scholz et al. 2014). Inversely, media portraying men as being proactive in managing their depression and open to confiding in others can help to de-stigmatize men’s depression (Scholz et al. 2014).

In terms of stigma and male suicide, men were found to hold more stigmatized beliefs than women about those who died by suicide (Batterham et al. 2013a). That said, Dahlen and Canetto (2002) reported that men tend to have more accepting attitudes toward male peers who consider suicide compared to females. Oliffe et al. (2011) argued that stigma invoked on family survivors of male suicide was potentially protective against self-harm for older men experiencing depression and suicidal ideation. Among young Irish men who had lost someone to suicide, there was a tendency to convey stigma around help-seeking for suicidal ideation, a desire to independently overcome such issues, and the perception that they themselves would be fragile if they had a mental illness and were to seek help (Butler and Phelan 2005).

Missing in the literature are Canadian perspectives regarding social and self-stigma associated with male depression and suicide. Moreover, sex differences in stigma specifically related to men’s depression and suicide are poorly understood. To address these knowledge gaps, we conducted a nationally based Canadian survey to assess stigmatic views, addressing whether such views differed as a function of respondents’ sex.

## Methods

The Behavioral Research Ethics Review Board at the University of British Columbia (H14-01991) approved this study.

### Recruitment Procedures

Participants were recruited via an online panel provider (Research Now Canada) and screened to ensure they met survey eligibility requirements (18 years and older, had online access, and were able to read English). The panel invitation did not disclose the survey topic, and only the 2108 potential respondents who went to the survey introduction page were advised that men’s depression and suicide were the focus. Of the 2108 potential respondents who went to the introduction page, 311 (14.8 %) answered ‘no’ to the opt-in, and 1797 answered ‘yes’ to opt in (and this was further reduced to 901 by post opt-in screening/quotas etc.). Respondents received honorarium points from the panel provider, which could later be exchanged for various rewards. To guard against the possibility of duplicate responses given the rewards, the respondents IP addresses were monitored and limited to single responses. The 8-min online survey was administered between August 29 and September 11, 2014.

### Survey Instruments

#### Depression Stigma Scale (DSS)

The Depression Stigma Scale (DSS) (Griffiths et al. 2004) was included in the survey protocol to assess social stigma, and completed by respondents without direct personal experience of depression. Using a 5-point Likert scale (strongly agree, agree, neither, disagree, strongly disagree), participants responded to 9 statements about men with depression, such as acceptance of depression as a mental health concern, beliefs around being able to control one’s depression, perceptions of danger, and potential neglect toward depressed men (Griffiths et al. 2004). The statements were modified to explicitly refer to men because the original items were gender neutral. For example, the statement “People with depression are dangerous” was modified to read “Men with depression are dangerous”. The DSS has been reported to have high internal consistency (Griffiths et al. 2004, 2008).

#### Stigma of Suicide Scale (SOSS)

The survey protocol also included the 16-item Stigma of Suicide Scale (SOSS) (Batterham et al. 2013b) to assess social stigma related to men’s suicide, and was completed by respondents without direct personal experience of depression or suicide. The SOSS utilizes a series of prejudicial terms (i.e., cowardly, stupid) to describe an individual who died by suicide. Respondents indicated their degree of agreement or disagreement to the descriptors using a 5-point Likert scale (strongly agree, agree, neither, disagree, and strongly disagree). Internal consistency of the SOSS is high (Chan et al. 2014).

#### Self-Stigma of Depression Scale (SSDS)

The Self-Stigma of Depression Scale (SSDS) (Barney et al. 2010) was included in the survey protocol to assess the degree of self-stigma related to depression among respondents who had direct personal experience with depression or suicide (defined as personally experiencing depressive symptoms or suicidal ideations or behaviors). The original version of the SSDS comprises 16-items classified into 4 subscales: shame, self-blame, help-seeking inhibition, and social inadequacy. We modified the scale by eliminating the following 4 items that were somewhat repetitive; “I would feel embarrassed; I would feel inferior to other people; I would feel I should be able to cope with things, and I would feel I couldn’t contribute much socially.” Using a 5-point Likert scale (strongly agree, agree, neither, disagree, and strongly disagree), participants responded to the remaining 12 statements. Test–retest reliability and internal consistency of the SSDS are high (Barney et al. 2010).

### Data Analysis

The proportions of the sample are reported that endorsed (‘strongly agree’ or ‘agree’ ratings) the individual items on the DSS, SOSS, and SDSS, which represent stigmatizing attitudes. Data weightings based on the 2011 Canadian Census for age, sex, and province (Statistics Canada 2013) were applied to correct for over/under sampling and to provide proportional representation to the survey findings. Chi square tests were used to identify statistically significant differences between male and female respondents at 95 % confidence using the Statistical Package for the Social Sciences (SPSS—Version 23).

#### Sample

A total of 901 English-speaking Canadian men and women completed the anonymous online survey. Participants resided in all regions of the country (Western Canada n = 456; Eastern Canada n = 445) and ranged in age from 18 to 83 years-old (mean = 50.5 years old). The sample was stratified and weights were employed to balance demographics, ensuring that the sample composition reflected the general Canadian population as determined by Census data. While sampling error cannot be estimated for non-probability samples such as ours, a traditional unweighted probability sample of comparable size would have produced results considered accurate to within plus or minus 4.6 percentage points, 19 times out of 20.

Sixty percent of the sample (N = 541; 281 men, 260 women) indicated ‘no direct personal experience’ with depression or suicide, and therefore completed the DSS and SOSS questionnaires. Forty percent of the total sample (N = 360; 171 men, 189 women) indicated ‘direct personal experience’ of depression and/or suicidal behaviors and completed the SSDS questionnaire.

## Results

### Social Stigma: Depression

**Fig. 1:**
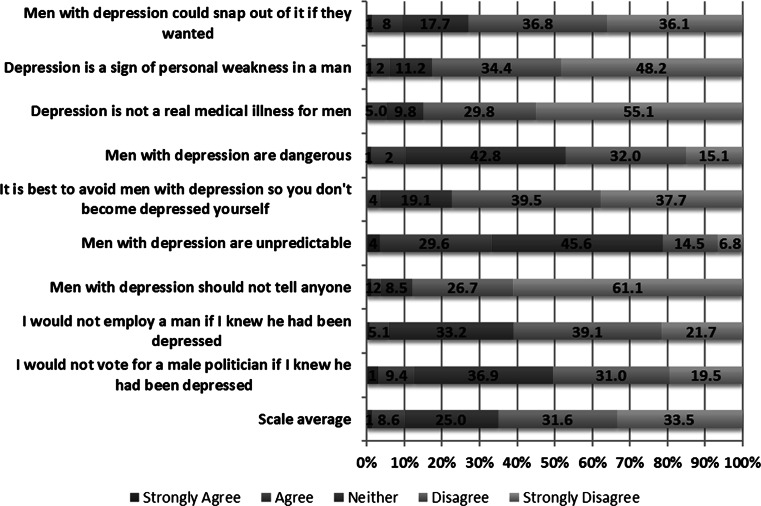
DSS—Overall item responses. “Using the scale below, please indicate how much you agree or disagree with the following statements about men’s depression”

Overall, there was minimal endorsement of items on the Depression Stigma Scale (DSS) by the 541 respondents who reported no personal experience with depression or suicide (see Fig. 1). The one exception concerned the item, “Men with depression are unpredictable”; 33.1 % of respondents (N = 179) endorsed this item (i.e., responded ‘strongly agree’ or ‘agree’). In contrast, respondents tended to renounce stigmatizing attitudes toward depression in men (i.e., responded ‘strongly disagree’ or ‘disagree’ to the items on the DSS). For example, 87.8 % of respondents (N = 475) opposed the item, “Men with depression should not tell anyone”. Similarly, most respondents disaffirmed the items, “Depression is not a real illness for men” (84.9 %; N = 459) and “Depression is a sign of personal weakness in a man” (82.6 %; N = 447).

Comparison of male and female responses to the DSS revealed several statistically significant differences, with a higher proportion of males endorsing stigmatizing attitudes on all but one item (see Table 1). The items with the largest differences between male and female respondents were, “I would not vote for a male politician if I knew he had been depressed”; “Men with depression are dangerous”; and “Men with depression could snap out of it if they wanted”. One item stood out as showing minimal difference in endorsement between male and female respondents, “Men with depression are unpredictable”; approximately a third of male and female respondents endorsed this item.

**Table 1:** DSS Sex Comparison

	% Agree^a^			
	Males (%)	Females (%)	Χ^2^	*p* value
Men with depression could snap out of it if they wanted	13.6	4.6	13.047	.000
Depression is a sign of personal weakness in a man	9.9	1.9	15.110	.000
Depression is not a real medical illness for men	9.4	0.8	19.769	.000
Men with depression are dangerous	15.1	4.5	16.504	.000
It is best to avoid men with depression so you don’t become depressed yourself	5.2	1.9	3.973	.046
Men with depression are unpredictable	34.1	31.9	3.119	.577
Men with depression should not tell anyone	5.9	1.2	8.593	.005
I would not employ a man if I knew he had been depressed	9.6	1.8	14.867	.000
I would not vote for a male politician if I knew he had been depressed	19.1	5.1	24.130	.000

^a^Based on combining “strongly agree” and “agree” responses

### Social Stigma: Suicide

**Fig. 2:**
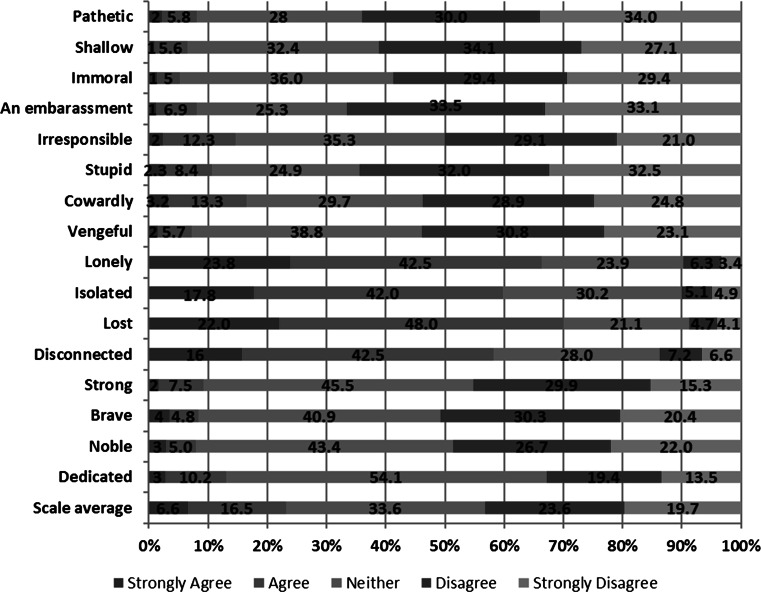
SOSS—Overall item responses**. “**Using the scale below, please rate how much you agree or disagree with the descriptions of men who take their own lives (suicide). In general, men who suicide are”

Generally, there were low levels of endorsement of stigmatizing items on the Stigma of Suicide Scale (SOSS) by the 541 respondents who reported no personal experience with depression or suicide (see Fig. 2). However, four items stood out in terms of drawing endorsement (strongly agree or agree) from a high proportion of respondents: “In general, men who suicide are…” Lost (70 %; N = 379), Lonely (66.3 %; N = 359), Isolated (59.8 %; N = 324), and Disconnected (58.5 %; N = 315). There were also a number of items representing stigmatizing attitudes that a large percentage of respondents disagreed with: “In general, men who suicide are…” An embarrassment (66.6 %; N = 360), Stupid (64.5 %; N = 349), Pathetic (64 %; N = 346), and Shallow (61.2 %; N = 331).

Comparison of male and female responses to the SOSS revealed statistically significant differences on 69 % of the items (see Table 2). In terms of specific items, greater proportions of female than male respondents endorsed items indicating that men who suicide are Disconnected (67.8 vs. 49.8 %), Lost (78.8 vs. 62.4 %), Lonely (73 vs. 60.3 %), and Isolated (63.8 vs. 56.2 %). For all other items showing significant differences, there were greater proportions of male respondents endorsing the stigmatizing items than female respondents.

**Table 2:** SOSS sex comparison

	% Agree^a^			
	Males (%)	Females (%)	X^2^	*p* value
Pathetic	12.0	3.3	13.919	.000
Shallow	8.8	3.8	5.510	.034
Immoral	8.9	1.1	16.638	.00
An embarrassment	11.1	4.8	7.211	.007
Irresponsible	18.0	10.8	5.612	.015
Stupid	15.1	5.5	13.186	.000
Cowardly	19.2	13.5	3.227	.081
Vengeful	11.4	2.5	16.204	.000
Lonely	60.3	73.0	9.828	.002
Isolated	56.2	63.8	3.262	.079
Lost	62.4	78.8	17.427	.000
Disconnected	49.8	67.8	18.189	.000
Strong	10.0	8.5	0.358	.569
Brave	8.6	8.1	0.037	.878
Noble	9.6	6.1	2.264	.158
Dedicated	16.7	9.0	7.093	.010

^a^Based on combining “strongly agree” and “agree” responses

### Self Stigma: Depression

**Fig. 3:**
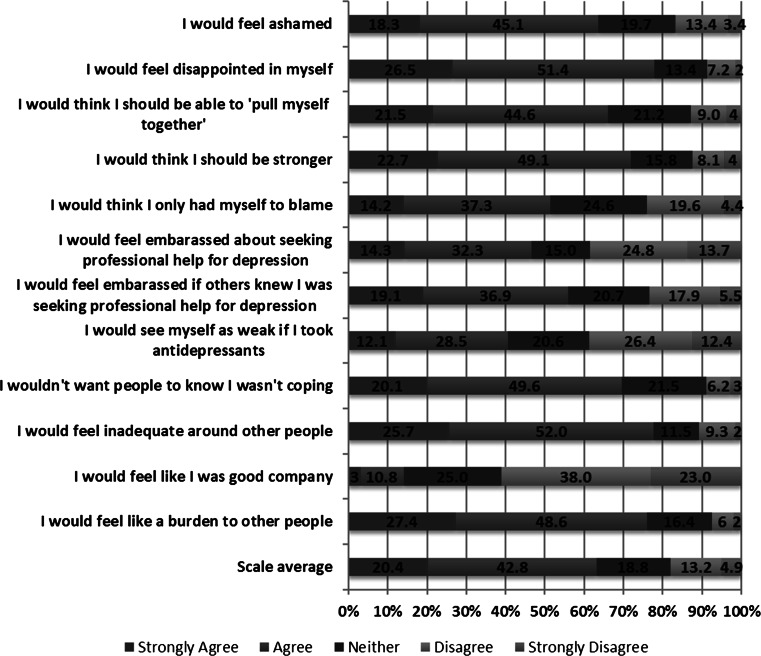
SSDS—Overall item responses. “Using the scale below, please indicate how much you agree or disagree with the following statements about depression. If I was depressed”

Amongst the sample of 360 respondents who indicated direct personal experience with depression and/or suicidal behaviors, there were high levels of endorsement of items on the Self-Stigma of Depression Scale (SSDS) (see Fig. 3). More than three-quarters of respondents endorsed the following items: “I would feel disappointed in myself” (77.9 %; N = 281), “I would feel inadequate around other people” (77.7 %; N = 280), and “I would feel like a burden to other people” (76.1 %; N = 274). All but two of the remaining items were endorsed by more than 50 % of respondents.

With regard to sex differences, male and female respondents differed significantly on one-third of the items on the SDSS (see Table 3). A greater proportion of male respondents, compared to female respondents, endorsed the following items: “I would feel embarrassed about seeking professional help for depression” (56.6 vs. 39.4 %) and “I would feel embarrassed if others knew I was seeking professional help for depression” (63 vs. 50.9 %). The reverse-scored item, “I would feel like I was good company”, drew more endorsement from female respondents than male respondents (66.4 vs. 53.3 %). A greater proportion of female respondents compared to male respondents also endorsed the item, “I would feel inadequate around other people”, (82 vs. 71.6 %).

**Table 3:** SSDS sex comparison

	% Agree^a^			
	Males (%)	Females (%)	X^2^	P-Value
I would feel ashamed	67.2	60.7	1.591	.224
I would feel disappointed in myself	75.5	79.6	0.882	.370
I would think I should be able to ‘pull myself together’	66.5	65.8	0.021	.910
I would think I should be stronger	67.7	74.7	2.136	.190
I would think I only had myself to blame	50.6	52.0	0.076	.749
I would feel embarrassed about seeking professional help for depression	56.6	39.4	10.289	.002
I would feel embarrassed if others knew I was seeking professional help for depression	63.0	50.9	5.108	.024
I would see myself as weak if I took antidepressants	44.3	37.9	1.444	.231
I wouldn’t want people to know I wasn’t coping	69.7	69.6	0.000	1.000
I would feel inadequate around other people	71.6	82.0	5.507	.029
I would feel like I was good company^b^	53.3	66.4	6.301	.016
I would feel like a burden to other people	77.3	75.1	0.223	.707

^a^Based on combining “strongly agree” and “agree” responses

^b^Based on combining “strongly disagree” and “disagree” responses

## Discussion

This national survey is the first in Canada to examine stigma (social and self) toward men with depression and men who suicide. Among respondents with no personal experience of depression or suicide (i.e., behaviors, ideations), there was minimal endorsement of stigmatizing attitudes toward men with depression. Instead, respondents tended to renounce stigmatizing views, as evidenced by the high proportion of participants who disagreed with statements such as “Men with depression should not tell anyone”; “Depression is not a real illness for men”; and “Depression is a sign of personal weakness in a man”. While a greater proportion of male respondents endorsed stigmatizing attitudes compared to female respondents, a finding consistent with previous research (Cook and Wang 2010; Wang et al. 2007a), they represented only a small minority of the total sample of men who responded. In contrast to the generally low support of stigmatizing attitudes, a third of respondents endorsed—with no significant difference between male and female respondents—a particular notion regarding men with depression: “Men with depression are unpredictable”. Previous studies have reported a similar finding (Cook and Wang 2010; Wang and Lai 2008). Our data do not permit us to elucidate why so many of the respondents endorsed this very particular outlook on men with depression, but it is tempting to speculate that media portrayals of men who commit violent crimes may feed such a perspective held by so many respondents (Oliffe et al. in Press). Though a previous Canadian study on social stigma around depression (Cook and Wang 2010) found higher endorsement of stigmatizing attitudes than in our survey, theirs was limited to a single province, to those with direct personal experience of depression or suicide, and posed questions to respondents in a gender neutral frame (i.e., a person with depression, as opposed to a man). Such differences make comparison of findings between studies tenuous.

Endorsement of stigmatizing attitudes toward men who take their own lives (suicide) was also generally low among respondents without a personal history of depression or suicidal ideation or behaviors. Rather, a considerable proportion of respondents rejected stigmatizing perspectives of men who die by suicide, disagreeing that such men can be described as “stupid”, “pathetic”, “shallow”, or “an embarrassment”. Though a greater proportion of male respondents, compared to female respondents, endorsed most of the items representing stigmatizing views of men who die from suicide, they were nevertheless a minority voice amongst male respondents. Standing out in contrast to these findings was the endorsement from the majority of respondents of a cluster of adjectives describing men who die from suicide—“lost”, “lonely”, “isolated”, and “disconnected”—suggesting that such men are socially and emotionally detached from others. Interestingly, a greater proportion of female respondents endorsed these items than male respondents, perhaps implying that the female respondents perceived a lack of connectedness to other people as a critical factor in male suicide.

Among respondents reporting direct personal experience with depression and/or suicidal behaviors, there was strong endorsement of stigmatizing attitudes toward one’s self about being depressed. Indeed, all but two items were endorsed by more than 50 % of the respondents, and more than 75 % of respondents affirmed that “I would feel disappointed in myself”, “I would feel inadequate around other people”, and “I would feel like a burden to other people”. For the most part, there were no significant differences in the proportions of male and female respondents endorsing self-stigmatizing views, highlighting that both males and females who suffer from depression struggle with internalized negative beliefs that likely contribute to their adverse emotional state (Mackenzie et al. 2004). However, a few statistically significant differences stood out. A greater proportion of male respondents, compared to female respondents, indicated that they would be embarrassed about seeking help for depression. Such findings may offer some insight as to why men are especially hesitant to seek mental health care, and or to disclose their help-seeking to others. Among the various factors influencing help-seeking by those experiencing depression, it seems that apprehension in having to speak out about one’s condition and fears around confidentiality serve as the most critical considerations and contributors to self-stigma (Clement et al. 2015). We also found that a greater proportion of female versus male respondents endorsed stigmatizing views of themselves as socially inadequate if depressed.

In terms of practical implications, given that a greater proportion of male respondents (compared to females) tended to endorse stigmatizing views of male depression and suicide, there is a need for health messaging and programs to target men in gender-sensitive and specific ways. For example, the permission and affirmation of other men can garner sustainable change in men’s health beliefs and behaviors (Oliffe et al. 2012). Therefore, reworking masculine ideals of self-reliance, strength and control toward disclosing and addressing male depression and/or suicidal thoughts might aid de-stigmatizing efforts and norm men’s mental health help-seeking. Avenues to achieving this might include anti-stigma workshops similar to those detailed by Michaels et al. (2014) but with a focus on male depression and suicide. Working with school age children as previously described by Ke et al. (2015) could also be adapted to focus on boys to reduce stereotypical beliefs about men’s mental illness and affirm help-seeking as a wise course of action.

Study limitations include the fact that we have drawn conclusions about sex differences without primary empirical evidence to describe how gender influenced the current study findings. To remedy this, future studies might include mixed methods to integrate sex and gender analyses as a means to thoughtfully considering men-centered interventions aimed at reducing male depression and advancing targeted suicide prevention efforts. Important additional findings may have also been garnered by including the SOSS questionnaire for respondents who had direct personal experience with depression or suicide. Balancing these limitations, the current study provides much needed insights to men’s depression and suicide stigma with a large representative Canadian sample.

The current study reveals stigma in male depression and suicide mostly among people with direct experience of depression/suicidal ideations as flowing from specific items that vary by sex and are deeply implicated in the discordant relationship between men’s low rates of diagnosed depression and high suicide rates. In this regard, targeted de-stigmatizing efforts can be reasonably argued as fundamental to raising public awareness and effectual self-management and lobbying effective services and policy action to reduce male depression and suicide.
